# Validation of the Thai version of the Female Genital Self-Image Scale (FGSIS)

**DOI:** 10.1186/s12905-022-01841-8

**Published:** 2022-06-25

**Authors:** Wanchat Komon, Athasit Kijmanawat, Kitti Chattrakulchai, Sirirat Sarit-apirak, Chatchawan Silpakit, Jittima Manonai

**Affiliations:** 1 Department of Obstetrics & Gynaecology, Faculty of Medicine Ramathibodi Hospital, Mahidol University, 270 Rama VI Road, Ratchathewi, Bangkok, 10400 Thailand; 2 Department of Obstetrics and Gynecology, Pramongkutklao Hospital and College of Medicine, Bangkok, Thailand; 3 Chakri Naruebodindra Medical Institute, Faculty of Medicine Ramathibodi Hospital, Mahidol University, Bangkok, Thailand; 4 Somdech Phra Debaratana Medical Center, Faculty of Medicine Ramathibodi Hospital, Mahidol University, Bangkok, Thailand; 5 Department of Psychiatry, Faculty of Medicine Ramathibodi Hospital, Mahidol University, Bangkok, Thailand

**Keywords:** Thai Female Genital Self-Image Scale, Female genital self-image, Questionnaire validation

## Abstract

**Background:**

Female genital self-image is associated with sexual health, sexual behavior, and gynecologic health behavior. The Female Genital Self-Image Scale (FGSIS) is a simple, validated instrument that quantifies genital self-image in women. The study aim was to translate the original English FGSIS into Thai and test its psychometric properties among Thai-speaking women.

**Methods:**

A cross-sectional, psychometric study of sexually active women attending a health check-up clinic at a university hospital in Thailand was conducted. On a volunteer basis and convenience sampling, 90 sexually active women were recruited between December 2020 and January 2021. Translation and transcultural adaptation of the English FGSIS into Thai were performed. The validity and reliability of the Thai FGSIS were assessed by examining content validity, face validity, internal consistency, construct validity, and test–retest reliability. The content validity of the Thai FGSIS was evaluated by assessing missing values, and internal consistency was evaluated using Cronbach’s alpha. Scores on the FGSIS and the Female Sexual Function Index (FSFI) were compared to examine convergent validity (using Pearson correlations). Confirmatory factor analysis (CFA) was also conducted. Test–retest reliability was measured by re-administering the Thai FGSIS to the same group of respondents after a 2-week interval.

**Results:**

The final Thai FGSIS was developed and assessed by a panel of experts. Data were examined for 86 respondents with average age of 32.5 ± 9.11 years. Content validity assessed using the level of missing data demonstrated no missing items. The overall internal consistency was high (Cronbach’s alpha: 0.847). Strong correlations (r = 0.61–0.83) between FSFI and FGSIS total scores (*p* < 0.01) were demonstrated. In addition, five domains of sexual functioning and the FSFI total score showed high correlations ranging from r = 0.089 to r = 0.383 (*p* < 0.05), which confirmed convergent validity. CFA identified a two-factor structure for the Thai FGSIS. The test–retest reliability for 38 participants was 0.937 (*p* < 0.05).

**Conclusion:**

The Thai FGSIS was found to be a highly valid and reliable instrument with which to measure female genital self-image in Thai-speaking women.

## Introduction

Female genital self-image is defined as women’s subjective perceptions and feelings about different aspects of their genitals, such as appearance, odor, and functionality [1, 2]. Previous studies have demonstrated that FGSI is associated with sexual health, sexual behaviors, and sexual satisfaction [1, 3]. In particular, embarrassment about exposing parts of the body during sex is associated with low sexual pleasure and poor sexual function [2, 4]. Genital self-image is not only related to sexual function but is also associated with gynecologic health behavior [1, 5]. Negative perceptions of their own genitals may cause women to postpone essential pelvic examinations. Moreover, many women have negative perceptions or misperceptions of their genitals; such perceptions are related to appearance dissatisfaction and probably reflect life experiences and media influence [6]. Consequently, female elective genital cosmetic surgery is increasingly popular in Western countries, and stems from a desire to improve genital appearance and self-esteem [7–10].

Female sexual dysfunction occurs when one of the phases in the sexual response cycle is compromised. It may be caused by any disruptions of physiology, psychology, experiences, beliefs, and relationships. One of the most important psychological factors that has been shown to affect female sexual function is body image [11]. Body image is an important concept for examining satisfaction or dissatisfaction with a sexual relationship. For example, worrying about physical appearance leads to decreasing self-esteem, desire, and pleasure [6]. Therefore, researchers have recently focused on the studies on their genital image as one of the body components that is involved in female sexual concerns. Given the importance of sexual function and female genital self-image on quality of life, it is essential to develop and validate scales to measure women’s genital self-perceptions. The Female Sexual Function Index (FSFI) is a reliable and valid measure of sexual functioning in women that focuses on female sexual arousal disorder [12] and hypoactive sexual desire disorder [13]. However, this instrument does not measure other aspects of female sexual dysfunction such as genital self-image [14]. The Genital Self-Image Scale-20 (GSIS-20) measures genital body image in women seeking treatment for sexual dysfunction [15]. It comprises 20 questions and has demonstrated high reliability and validity across several populations of women. The Female Genital Self-Image Scale (FGSIS) was developed and validated in 2010 to quantify genital self-image in women [1–3]. This scale comprises seven items that assess a broader spectrum of female genital self-image, including women’s perceptions of their genitals and their feelings when having genital or pelvic examinations [1]. The psychometric properties of the FGSIS have been confirmed across various U.S. general populations and college samples [2, 5]. Given that women’s sexuality is heavily affected by cultural factors, this scale has been successfully validated for use in the Iranian, and Turkish population [16, 17]. Besides the strong psychometric properties to assess perceived female genital image, the FGSIS is also easy to use in wide-ranging population.

There is a need for an appropriate instrument with which to assess genital self-image in women in Thailand in order to be reliably used in genital cosmetic surgeries and in a variety of gynecologic conditions. We therefore searched for the Thai version of the FGSIS which demonstrates feasibility, validity, and reliability for use with Thai women. After we conducted a literature review and sought permission to use the original and Thai FGSIS and found that the officially validated questionnaire in Thai language was not available. Therefore, the aim of the present study was to translate the original English FGSIS into Thai and to test its psychometric properties among Thai-speaking women.

## Methods

### Setting

This study was conducted at the outpatient clinic at the Department of Obstetrics and Gynaecology, Faculty of Medicine Ramathibodi Hospital, Mahidol University, Bangkok, Thailand, between December 2020 and January 2021. The validation procedure comprised two phases: (1) translation and (2) testing of psychometric properties.

### Phase 1 translation

Transcultural adaptation of the original English FGSIS into a Thai version was carried out according to the process for patient-reported outcome measures [18]. We began the translation process after obtaining written permission to use the FGSIS from the developer of the scale, Debra Herbenick.

1. Two Thai-speaking translators experienced in translating health questionnaires independently conducted forward translation of the FGSIS into Thai.
2. An expert experienced in the questionnaire validation process (CS) compared and combined the two forward translations into a reconciled Thai version.
3. Two bilingual native English speakers, who had not seen the original version of the scale and were unaware of the study purpose, individually translated the reconciled Thai version back into English.
4. The two back translations were then reviewed by two gynecologists (WK and AK), one nurse (SS), and the questionnaire validation expert (CS) to ensure the accuracy of translation. Any misunderstandings or unclear expressions in the translated versions were examined and revised to produce a final Thai version.

### Phase 2 psychometric property testing

The validity and reliability of the Thai FGSIS were assessed in a cross-sectional, psychometric study of women attending a health check-up clinic during the study period. On a volunteer basis and non-probability sampling method, eligible participants were recruited according to the inclusion and exclusion criteria. Eligible participants were sexually active women (who had engaged in any type of sexual relations at least once in the previous month) who were willing to participate, were able to communicate fluently in Thai, and who provided written informed consent. Participants were excluded from the study if they withdrew or were unable to complete the questionnaire. No compensation of any type was provided.

The final version of the Thai FGSIS was evaluated according to the COnsensus-based Standards for the selection of health Measurement INstruments (COSMIN) methodology for evaluating the content validity of patient-reported outcome measures [19]. The psychometric properties examined were content validity, face validity, internal consistency, construct validity, and test–retest reliability. Details of the psychometric testing stages are as follows.

1. Content validity (including face validity) was assessed according to the COSMIN methodology [19]. The final draft of the questionnaire was tested on a heterogeneous group of twenty Thai laypeople women. Participants were asked to complete the final draft of the questionnaire and provide feedback on any incomprehensible item words, the difficulty level, and the cultural relevance of the translation. They could discuss any general or specific questions with the principal investigator (WK), who observed the volunteers completing the questionnaire, and controlling the pilot procedure. Upon completion, the participants were briefly interviewed about the meaning of each item. In parallel, five obstetricians & gynecologists and one psychiatrist were asked for a clinician’s review of the final draft questionnaire. Furthermore, levels of missing data were also measured and used as an indicator of inappropriate items. Results of the pilot testing were taken into consideration when producing the final version of the Thai FGSIS used in the study. Since content validity of the original FGSIS questionnaire has been demonstrated previously [1], this study only tested the interpretability of the items in terms of questionnaire completion time [19].
2. Internal consistency or reliability was evaluated by examining the correlations between the questionnaire items. Cronbach’s alpha coefficient and item—total correlations were calculated to assess the consistency of the test items.
3. We assumed that genital self-image is closely associated with sexual functioning. Therefore, we predicted that genital self-image scores would correlate positively and highly with sexual functioning scores. We compared scores on the FGSIS and FSFI to examine the construct validity of the Thai FGSIS. Convergent validity was used as a supporting piece of evidence for construct validity.
4. Test–retest reliability is a measure of the stability of survey scores. The stability of the Thai FGSIS was measured by administering the questionnaire to the same group of respondents, with a 2-week interval between the initial and second tests. All participants were invited to participate in the retest.

### Instruments

The FGSIS was methodically developed, and psychometric testing has shown that the scale has good validity and reliability [2, 3]. The FGSIS comprises seven items that assess women’s feelings and beliefs about their own genitals. Responses are on a 4-point descending scale (strongly agree, agree, disagree, strongly disagree). Respondents’ scores on each item are summed to obtain a total score (range 7–28). There is no cut-off point; higher scores indicate more positive genital self-image [1].

The FSFI is a self-report questionnaire developed to measure female sexual function over the previous 4 weeks. It consists of 19 items that encompass six separate domains: desire, arousal, lubrication, orgasm, satisfaction, and pain. Response scores range from 1 to 5; higher scores indicate better sexual functioning [12, 13]. The FSFI has been widely used and translated into several languages, including Thai [20].

### Ethical considerations

The study was approved by the human research ethics committee of the Faculty of Medicine, Ramathibodi Hospital, Mahidol University (MURA 2020/1777). All participants signed an informed consent form before being study enrolment. Permission was obtained from the original author of the FGSIS to translate the scale into Thai. All methods were carried out in accordance with the Declaration of Helsinki for medical research involving human subjects.

### Statistical analyses

The demographic and baseline characteristics of the study population were described as mean and standard deviation (SD) or number and percentage for categorical data. The content validity of the Thai FGSIS was evaluated using the pattern of missing values, and internal consistency was determined using Cronbach’s alpha [21]. Convergent validity of the Thai-FGSIS was examined by Pearson correlations between the total scores of the Thai-FGSIS and six domains of sexual functioning and the FSFI. Furthermore, the factor structure was examined with confirmatory factor analysis (CFA) and used to determine the construct validity of the Thai-FGSIS [22–24]. A series of CFAs were carried out to investigate how well the model fit the observed data. CFA was conducted with maximum likelihood estimation on all seven questionnaire items. A combination of fit indices was used to assess the overall fit of the model including chi-square (χ^2^) goodness-of-fit statistic, comparative fit index (CFI), Tucker–Lewis index (TLI), root mean square error of approximation (RMSEA), and standardized root mean square residual (SRMR). Test–retest reliability was assessed by comparing the mean test–retest scores for each domain using intraclass correlation coefficients [25].

Test results were considered statistically significant at *p* < 0.05. Data were analyzed using IBM SPSS Statistics for Windows, version 27.0 (IBM Corp., Armonk, NY, USA) and Analysis of Moment Structures (AMOS) 26.0.0 (IBM, SPSS Inc., USA).

### Sample size estimation

To satisfy the 10 respondents-per-item ratios as recommended for the required sample size in a validation study [26], validation of a questionnaire with seven items requires a sample size of 70. Therefore, the required sample size was estimated as 84 to allow for a 20% dropout rate.

## Results

### Phase I: translation

After completion of the translation and cross-cultural adaptation process, the panel of experts agreed that the final Thai translation was understandable and evaluated the intended concepts. The Thai FGSIS was then assessed with a small group of 20 volunteers. Pilot testing and cognitive debriefing interviews were conducted. The volunteers confirmed that the final draft of the scale was feasible; the items were clear, unambiguous, and easy to understand and respond to; and the items were culturally relevant.

### Phase II: instrument validity and reliability assessment

The respondents comprised 86 women. Initially, 90 women participated in this study, but 4 participants were lost because they did not return the questionnaire. The response rate was 95.6%. Table 1 shows the demographic characteristics of the respondents. The mean age was 32.5 years (SD = 9.11, range 20–54 years). Most participants were single, primiparous, premenopausal, and had at least a bachelor’s degree.

**Table 1:** Respondent demographics (N = 86)

Variable	N	%
*Marital status*		
Single	51	59.3
Married	34	39.5
Divorced	1	1.2
*Parity*		
Nulliparous	53	61.6
Parous	33	38.4
*Menopausal status*		
Premenopausal	79	91.9
Postmenopausal	7	8.1
*Education level*		
High school or lower	26	30.2
Bachelor’s degree or higher	60	69.8

### Content and face validity

The results of the content validity testing showed that all Thai FGSIS items were appropriate, clear, and culturally relevant. All respondents took less than 2 min to complete the 7-item, self-administered Thai FGSIS, with a mean of 55.2 ± 22.8 s. In addition, there weren’t missing items among 86 responses. The content and face validity of the Thai FGSIS proved good interpretability of the items.

### Internal consistency (reliability)

The mean Thai FGSIS score was 21.72 (SD = 4.17; N = 86), indicating relatively high genital self-image; scores ranged from 10 to 28. Table 2 shows the results of the internal consistency assessment of the Thai FGSIS. Cronbach’s alpha was 0.847 (95% confidence interval, 0.545 to 0.980), indicating good reliability. The sequential removal of items did not significantly change the alpha value.

**Table 2:** Internal consistency of the Thai version of the Female Genital Self-Image Scale (N = 86)

FGSIS item no	Mean	Standard deviation	Corrected item, total correlation (*r*)	Cronbach’s alpha if item deleted	Factor loadings
1	3.31	0.16	0.704	0.817	0.824
2	3.28	0.66	0.772	0.808	0.866
3	2.85	0.96	0.641	0.822	0.763
4	3.05	0.85	0.447	0.850	0.582
5	3.45	0.55	0.560	0.837	0.676
6	2.99	0.94	0.556	0.836	0.663
7	2.80	1.02	0.703	0.811	0.802

FGSIS: Female Genital Self-Image Scale

### Construct (convergent) validity

Thai FGSIS scores correlated significantly with FSFI total scores and with scores on the five FSFI domains of sexual functioning. Correlations ranged from r = 0.089 to 0.383 (*p* < 0.05) (Table 3). Table 4 shows the correlations between FGSIS total scores and FGSIS item scores. These confirmed the reliability of the scale.

**Table 3:** Construct (convergent) validity of the Thai version of the Female Genital Self-Image Scale (N = 86)

	1	2	3	4	5	6	7	8
1. FGSIS-7	–	0.313^**^	0.383^**^	0.260^*^	0.261^*^	0.287^**^	0.089	0.350^**^
2. FSFI desire		–	0.787^**^	0.649^**^	0.449^**^	0.557^**^	0.289^**^	0.681^**^
3. FSFI arousal			–	0.823^**^	0.610^**^	0.702^**^	0.407^**^	0.792^**^
4. FSFI lubrication				–	0.746^**^	0.763^**^	0.537^**^	0.811^**^
5. FSFI orgasm					–	0.704^**^	0.515^**^	0.724^**^
6. FSFI satisfaction						–	0.557^**^	0.829^**^
7. FSFI pain							–	0.634^**^
8. FSFI total scale								–

*p < 0.05, **p < 0.01. FGSIS: Female Genital Self-Image Scale; FSFI: Female Sexual Function Index

**Table 4:** Intercorrelations among the Thai version of the Female Genital Self-Image Scale items (N = 86)

FGSIS item no	1	2	3	4	5	6	7	FGSIS total score
1	–	0.85^***^	0.54^***^	0.43^***^	0.49^***^	0.39^***^	0.49^***^	0.78**
2	–	–	0.59^***^	0.41^***^	0.46^***^	0.50^***^	0.59^***^	0.83**
3	–	–	–	0.27^*^	0.42^***^	0.41^***^	0.67^***^	0.78**
4	–	–	–	–	0.43^***^	0.28^**^	0.35^***^	0.61**
5	–	–	–	–	–	0.33^**^	0.45^***^	0.65**
6	–	–	–	–	–	–	0.58^***^	0.70**
7	–	–	–	–	–	–	–	0.83**

*p < 0.05, **p < 0.01, ***p < 0.001. FGSIS: Female Genital Self-Image Scale

**Fig. 1:**
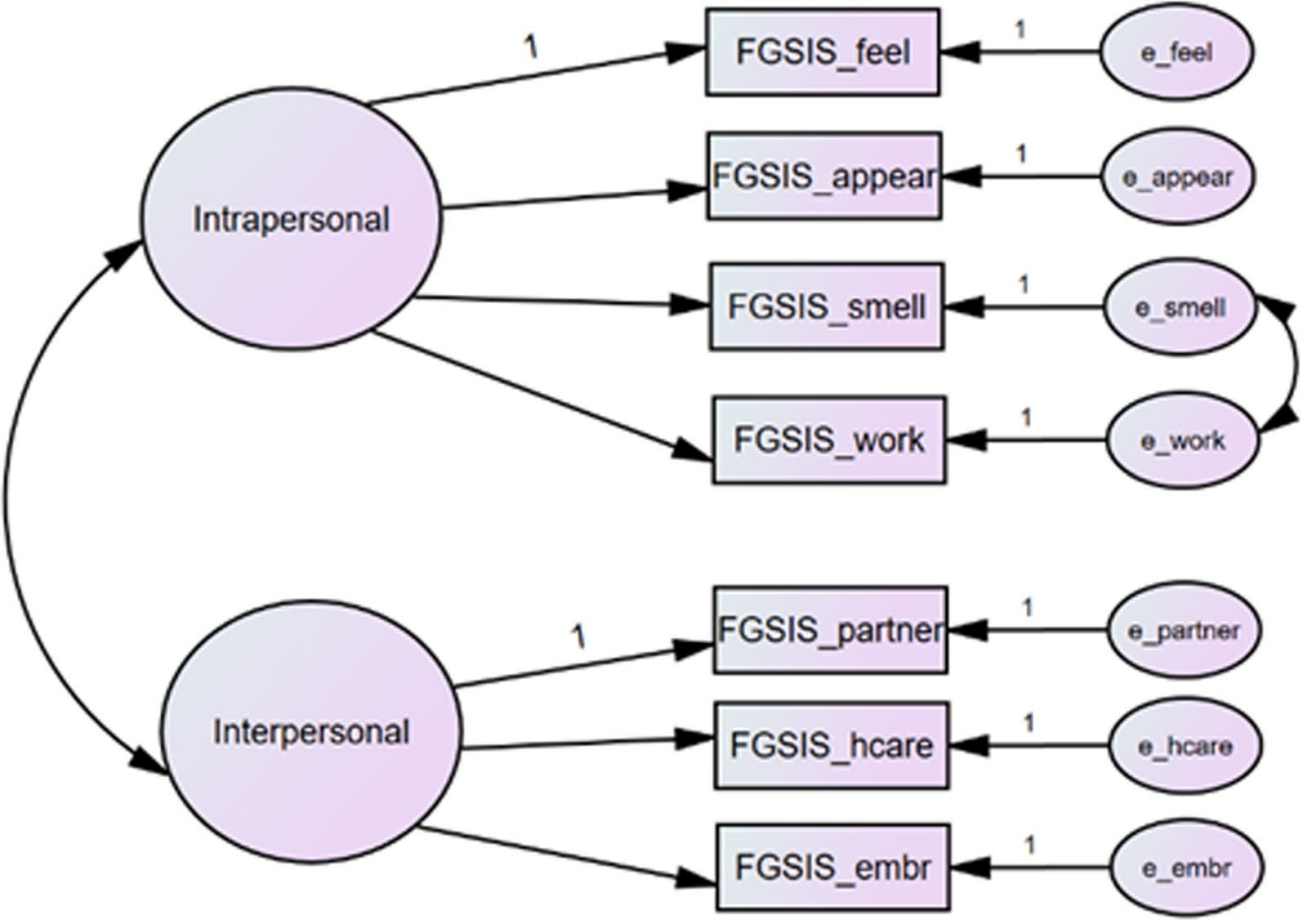
Two-factor confirmatory factor analysis of the Thai version of the Female Genital Self-Image Scale (Thai FGSIS). feel: I feel positively about my genitals. appear: I am satisfied with the appearance of my genitals. smell: I think my genitals smell fine. work: I think my genitals work the way they are supposed to work. partner: I would feel comfortable letting a sexual partner look at my genitals. hcare: I feel comfortable letting a health care provider examine my genitals. embr: I am not embarrassed about my genitals

The original one-factor structure of the FGSIS could not be simulated by the CFA conducted on our sample. The fit indices for this model were: χ^2^ of 51.39 and degrees of freedom (*df*) of 14 at a probability of < 0.01, CFI = 0.87, TLI = 0.81, RMSEA = 0.18 and, SRMR = 0.08. The proposed two-factor model (Factor 1 = intrapersonal concerns; Factor 2 = interpersonal concerns) was assessed using CFA according to the recommendations of the authors of the original FGSIS. A final two-factor model was obtained in the second step, after examining the measurement invariance. The fit indices of the final two-factor model with covariance parameters (Fig. 1) showed a χ^2^ of 16.98 and 12 degrees of freedom; the probability = 0.151, comparative fit index = 0.98, Tucker–Lewis index = 0.97, root mean square error of approximation = 0.07, and standardized root mean square residual = 0.05. The resulting two-factor model demonstrated a better fit to the data. Table 5 shows fit statistics for the CFA model.

**Table 5:** Fit statistics for confirmatory factor analysis (CFA) of the Female Genital Self-Image Scale Thai version (N = 86)

Model	χ^2^	*df*	CFI	TLI	RMSEA (90% CI)	SRMR
Single factor	51.39	14	0.87	0.81	0.18 (0.13–0.23)	0.08
Two-factor	16.98	12	0.98	0.97	0.07 (0.02–0.16)	0.05

*χ*^*2*^ chi-square, *df* degrees of freedom, *CFI* comparative fit index, *TLI* Tucker–Lewis index, *RMSEA* root mean square error of approximation, *CI* confidence interval, *SRMR* standardized root mean square residual

### Test–retest reliability (stability)

For the 38 women who completed the first and second questionnaires, the overall test–retest reliability was 0.937 (Table 6). The test–retest correlations for each item were between 0.773 and 0.930, indicating good-to-excellent reliability.

**Table 6:** Test–retest reliability of the Thai version of the Female Genital Self-Image Scale (N = 38)

FGSIS item no	Test–retest correlation coefficient (*r*)	95% confidence interval	*p* value
1	0.930	0.869–0.963	0.00
2	0.902	0.820–0.948	< 0.01
3	0.866	0.757–0.928	< 0.01
4	0.921	0.853–0.958	0.00
5	0.789	0.630–0.885	< 0.01
6	0.883	0.786–0.937	< 0.01
7	0.773	0.604–0.875	< 0.01

*FGSIS* Female Genital Self-Image Scale

## Discussion

Female genital self-image is an important factor that affects sexuality, sexual behavior, and gynecologic health behavior [1–3, 5]. Its effects and contributing factors have been widely studied in developed countries [27] but poorly evaluated in women in developing countries. This could be explained by differences in socioeconomic and cultural conditions across countries, and by a lack of appropriate instruments to quantify this sensitive concept. A range of cultural, biological, and psychological factors may affect female genital self-image. Therefore, cultural adaptation and evaluation of the cross-cultural applicability of an appropriate measure is important. The present study aimed to determine the psychometric properties of a translated and culturally adapted version of the FGSIS. In summary, our findings demonstrated that the Thai FGSIS has high validity and reliability for use with Thai-speaking women. Moreover, the scale seems feasible for use in hospital or community settings because it is short and the items and response options are easy to understand. These factors may increase respondent engagement.

A questionnaire-based survey is considered highly reliable if the results are reproducible over time under the same conditions. Consistent with previous reports [1, 2, 16, 17], we found that the Thai FGSIS demonstrated high levels of reliability in terms of internal consistency and stability. The test–retest correlations for all items demonstrated that the questionnaire produced consistent responses. These excellent psychometric properties are similar to those demonstrated for the original English version [1] and the Iranian and Turkish versions [16, 17] of the FGSIS.

Factor analysis was used to examine the construct validity of the scale. The Thai FGSIS generated a one-factor model. The two-factor (intrapersonal and interpersonal) structure suggested by the original authors also sufficiently fit the model [3]. This flexibility should be analyzed in future studies. We found that measures of genital self-image assessed by the Thai FGSIS correlated highly with measures of sexual functioning assessed by the FSFI, with the exception of the FSFI pain domain. Therefore, our findings indicate the reliability and validity of the Thai version of the FGSIS for quantifying female genital self-image in Thai-speaking women. Because female genital self-image is culturally influenced, a fully validated and culturally adapted version of the FGSIS is useful to evaluate women’s genital self-perceptions. This scale could be used for surveys in clinical, research, and public health settings.

The present study could not prove the originally proposed unidimensional structure of the FGSIS but instead found evidence for a two-factor structure, consisting of one factor on interpersonal concerns and another factor assessing on intrapersonal concerns [1]. Possible explanations for the discrepancies in the factor structure could be different sample characteristics. The original validation study used a nationally representative sample of U.S. women aged 18 to 60 [2], whereas our study used a convenience sample of women with a relatively narrow age range (20 to 54 years), which could have affected the results and restricted comparability of the findings. Also, women included in our study were more highly educated in comparison to Herbenick et al. study [2]. Previous studies examined the extent to which multidimensional factors associated with more negative genital image [14, 27]. Women with higher education especially sexual health education may be more exposed to educational information via media and/or the Internet, which could shape their genital self-perceptions [27].

From a clinical perspective, women’s satisfaction or dissatisfaction with their genital appearance affects sexual function, self-esteem, and gynecological screening [1, 3, 5]. It can be difficult for women to address genital self-image and sexual concerns. In Eastern countries, women are traditionally reluctant to express such concerns; therefore, validated, culturally adapted instruments in their own language are useful to encourage openness about these topics. The Thai FGSIS contains only seven short and simple items. Data are quick and easy to collect using paper, online, or mobile versions of the scale. This type of scale is suitable for measuring private and sensitive issues and promoting comfortable and caring communication between women and their healthcare providers. Addressing these issues would help to identify and resolve misconceptions about genital appearance and the associated desire for unnecessary cosmetic surgery.

One of the strengths of this study was the reliable cross-cultural adaptation and the validation stage, which is crucial to ensure the psychometric properties. The testing of the Thai FGSIS used a sample size appropriate for the respondent-to-item ratios and a satisfactory response rate was obtained. As a result, this study provides good support for the validity of the FGSIS in Thai as well as evidence for the usefulness of this instrument for epidemiological research and clinical purposes in the target populations. There were also some study limitations that should be acknowledged. Because the sample was obtained from a health check-up center, the respondents were younger and had a higher level of education than the general population. This may reduce the generalizability of the findings. In addition, owing to the lack of a gold standard for measuring female genital self-image, we did not assess the criterion validity of the scale. Future studies should examine the Thai FGSIS with the general population and with women in other contexts. Studies are also needed on the responsiveness of the Thai FGSIS to investigate the effect of treatment on patients’ perceptions of their genitalia.

## Conclusions

The study findings indicate that the Thai FGSIS is a quick, valid, and reliable measure of female genital self-image in the Thai population.

## Data Availability

The datasets generated and/or analyzed during the current study are not publicly available due to the privacy of the subject but are available from the corresponding author on reasonable request.
